# How can we get more people with long-term health conditions involved in *parkrun*? A qualitative study evaluating *parkrun’s* PROVE project

**DOI:** 10.1186/s13102-019-0136-6

**Published:** 2019-10-17

**Authors:** Helen Quirk, Steve Haake

**Affiliations:** 1 Centre for Sport and Exercise Science, Sheffield Hallam University, Sheffield, S10 2BP UK; 2 Advanced Wellbeing Research Centre (AWRC), Sheffield Hallam University, Sheffield, UK

**Keywords:** Physical activity intervention, Evaluation, *parkrun*, Long-term health conditions, Disability, Volunteering

## Abstract

**Background:**

People with long-term health conditions face barriers to physical activity and community health interventions despite potential life-changing benefits for self-management of their condition and wellbeing. A weekly mass participation running, walking and volunteering event called *parkrun* launched a project called PROVE in 2016 to engage people living with long-term health conditions in England. Over the 3 year project, *parkrun* appointed volunteer Outreach Ambassadors with a specialist interest in the health condition they represented whose role was to ensure *parkrun* was welcoming, supportive and inclusive. This qualitative study aimed to understand the experience of the PROVE project for people with long-term health conditions.

**Methods:**

Semi-structured interviews were conducted with 15 PROVE Outreach Ambassadors representing 13 different long-term health conditions in England. Interviews were recorded, transcribed verbatim and analysed using thematic analysis. Rigour and transparency were sought in addition to utilising independent researchers to offer alternative interpretations of the data.

**Results:**

Data analysis resulted in 4 overarching themes and 13 subthemes. Outreach Ambassadors believed that *parkrun* was already supportive of people with long-term health conditions, but that the PROVE project enabled the support to be delivered in a more structured way across health conditions and locations. Outreach Ambassadors believed that the PROVE project had the potential to create a welcoming, safe space for people with long-term health conditions to participate as walkers, runners or volunteers. Success of the PROVE project was believed to be dependent on being realistic about the potential to bring about change, challenging people’s perceptions of *parkrun* and engaging with key stakeholders and advocacy groups. Challenges for *parkrun* were believed to be around communication, demonstrating impact and the project’s dependence on volunteers for delivery.

**Conclusions:**

This is the first study of its kind to explore the public health potential of *parkrun* for people with long-term health conditions. *parkrun’s* PROVE project was regarded to be important for ensuring that people with long-term health conditions can engage in physical activity and volunteering in a safe and supportive environment. The findings have important implications for *parkrun*, policy makers and physical activity providers looking to deliver inclusive community physical activity opportunities.

## Background

In the United Kingdom (UK) more than 26 million adults have at least one long-term health condition (LTC) such as diabetes, asthma, heart failure, arthritis, dementia or depression [1]. Supporting self-management among people with LTCs is important for people’s positive attitudes and behaviours, quality of life, clinical symptoms and use of healthcare resources [2]. Community-centred approaches for health offer opportunities for self-management practices that foster social inclusion, enhanced wellbeing and behaviour change, especially group activities with a focus of shared interest [3]. As one part of self-management, physical activity can be an effective way of promoting social inclusion and supporting people with LTCs to self-manage their condition, yet rates of participation by people living with a physical, intellectual or mental LTC are low compared to people not living with LTCs [4–6]. There is conclusive evidence that people with LTCs face substantial personal (e.g., pain, motivation) and environmental (e.g., architectural barriers and physical accessibility) barriers to engaging in active lifestyles [7, 8] and inclusive physical activity and volunteering opportunities that are appropriate for people with LTCs are urgently needed [9, 10].

Whilst community-based physical activity participation could be particularly important for promoting independence among adults with LTCs, a number of health inequalities (e.g., disability) exist that limit involvement for people living with LTCs. Those living with disabilities are less likely to be active compared to people without disabilities [11]. Effective, ‘real world’ community physical activity opportunities to tackle health inequalities are needed, especially those that involve all health condition groups. *parkrun* (*parkrun* is always written as one word with a lowercase ‘p’) is a community-based physical activity and volunteering event that recognises and supports the needs of people with LTCs and offers an opportunity for using the community to promote health and wellbeing. This research begins to explore the public health potential of *parkrun* for people living with LTCs.

Launched in 2004, *parkrun* (www.parkrun.com) is a rapidly growing mass participation event encouraging anyone over the age of 4 to either run, walk or wheel (buggy running and wheelchairs) 5 km, or volunteer their time to facilitate the weekly event. *parkrun* takes place every Saturday morning across 22 countries, is free and welcomes people of all backgrounds and abilities. Since 2010, junior *parkrun* 2 km events for 4–14 year olds and their families have been held on a Sunday morning. *parkrun* has a vision to create a “healthier, happier planet” [12] and aims to do so by striving to be as inclusive and welcoming as possible to people of all backgrounds and abilities. Anecdotally, *parkrun* is known for its community spirit and welcoming nature, and a growing body of research evidence supports the notion that *parkrun* is perceived as an inclusive community by those who take part [13–16].

Over 160,000 people walk, jog, run and volunteer at their local *parkrun* across more than 800 locations in the UK every weekend. Despite its inclusive nature and being one of the largest providers of physical activity in the UK, *parkrun* grew organically and was initially promoted via word of mouth. As such, certain groups and communities have been less well represented in the *parkrun* population [17], such as those living with LTCs and disabilities. In 2013, Stevinson and Hickson reported that just 4.3% of 7308 adult UK *parkrun* participants reported a limiting disability [18]. Recognising this, the *parkrun* management team implement targeted attempts to increase reach and engage underrepresented groups. An example of this was the PROVE project. The PROVE project (***p****arkrun*: **r**unning **o**r **v**olunteering for **e**veryone) was a 3 year project launched in 2016 to increase engagement in *parkrun* by people living with LTCs in England. This is the first manuscript to present initial findings from the evaluation of the PROVE project.

PROVE was based on a peer support approach, led by volunteer Outreach Ambassadors with no formal training requirements or qualifications, but with a specialist interest in the condition groups being targeted. Specialist interest could be in the form of personal, lived experience of the condition either as someone living with the condition or a carer for someone with the condition, or as a health professional working in the field of that condition. Outreach Ambassadors were recruited by *parkrun* from volunteers in the existing *parkrun* community. It was the role of volunteer Outreach Ambassadors to engage with the existing *parkrun* community as well as the wider community of people living with the LTC they represent, working with relevant organisations or advocacy groups to strengthen *parkrun*’s engagement with LTC populations. The Outreach Ambassadors had the responsibility of identifying the needs of people living with LTCs, designing and implementing interventions that aimed to facilitate engagement and support the positive experience of *parkrun* by those involved. It was one of the aims of the PROVE project to ensure that the support put in place for people living with LTCs could be delivered sustainably on *parkrun*’s model of being volunteer-driven.

Exploring the experience of physical activity from the perspective of those living with LTCs is important to understand how to foster physical activity environments and interventions that are appropriate, supportive, safe and welcoming for all. A study into *parkrun* by Morris and Scott (2018) [15] involved interviews with 20 *parkrun* participants in the UK who had a history of mental health conditions. This study found that participants experienced *parkrun* as inclusive and welcoming, a safe environment and the familiarity of *parkrun* was comforting. Participants felt part of a family, valued opportunities to socialise and make friends and to identify themselves as part of a community rather than as someone with a mental health condition. In this research, Morris and Scott (2018) have made important advances into uncovering the experience of *parkrun* for people living with health conditions. More research into a wider range of LTCs will enable *parkrun* and policy-makers to recognise and respond to the specific adaptations that must be embedded in communities in order to make physical activity safe and accessible for people with LTCs [19].

This qualitative study aims to understand the perceptions of *parkrun* and the PROVE project for people living with LTCs from the perspective of *parkrun* volunteer Outreach Ambassadors. The results will have direct implications for the wider evaluation of the PROVE project, but also implications for physical activity providers, public health practitioners, stakeholders and policy makers. This manuscript presents preliminary findings from the evaluation of the PROVE project, the final evaluation results will be disseminated in a later publication.

## Methods

The research methods were reviewed and approved by the local University Research Ethics Committee (reference number: HWB-2016-17-S&E-29).

### Participants

The current research sought to include Outreach Ambassadors from across the condition groups targeted by *parkrun* in the PROVE project. Outreach Ambassadors in England representing the following LTC groups were invited for an interview: dementia (including Alzheimer’s Disease), deaf and hard of hearing, blood pressure conditions, cerebral palsy, heart conditions, endometriosis, diabetes, learning disabilities and/or autism, obesity, asthma, multiple sclerosis, mental health, musculoskeletal conditions.

The rolling recruitment of Outreach Ambassadors occurred between March 2017 and August 2018 using a purposeful sampling procedure. Outreach Ambassadors who were within 2 months of being appointed to their role were invited via an email sent out by a member of *parkrun* staff with the research information sheet. In total, 33 Outreach Ambassadors were invited for an interview (the number of Outreach Ambassadors in role at the time of recruitment). Invitees were asked to contact the research team directly to express their interest in participating. Those willing to participate in a telephone interview signed a consent form electronically prior to the telephone call being arranged. Fifteen Outreach Ambassadors (45%) gave written consent to be interviewed. A mutually convenient interview time was arranged with the lead researcher on the evaluation team (HQ).

### Data collection

Data were collected by the lead researcher (HQ) via semi-structured interviews with *parkrun* Outreach Ambassadors across England. The interviewer was the lead researcher (HQ) who is trained in qualitative research. HQ is a female researcher in the field of exercise psychology with personal experience of *parkrun* as a runner, walker and volunteer. Interviews took place between March 2017 and August 2018 over the telephone and were recorded using a digital sound recording device and telephone pick-up. The interviewer aimed to create a free-flowing discussion directed by the interviewee in an informal conversational style. The questions used were open-ended and an interview schedule was used to ensure consistency across interviews. Interview questions included:

- *Can you describe your experience of parkrun?*
- *How does parkrun support people who have a LTC?*
- *What do you think about the PROVE project?*
- *What motivated you to get involved with the PROVE project?*
- *What would success look like to you? / What changes do you expect to see?*
- *What skills and qualities do you believe are important for your role as a parkrun Outreach Ambassador?*
- *What challenges do you foresee with your role as a parkrun Outreach Ambassador?*

One interview was conducted in written format via email (i.e., *asynchronous online interview* [20]) due to the participant being unable to communicate via telephone. The written format involved extracting questions from the interview guide and included any probes that the interviewer would have used in a spoken interview. Interview length ranged from 17 to 61 min and the mean interview duration was 36.4 min.

### Data analysis

Audio recordings of the interviews were transcribed verbatim by an external transcription company. Data were analysed thematically by the lead researcher (HQ). Thematic analysis [21] was an iterative process that involved familiarisation with the data (reading and re-reading), generation of initial codes,, searching for themes (grouping similar codes into themes), reviewing potential themes, defining and naming themes and sub-themes and finally writing up the themes. Data analysis started with an inductive approach (deriving primarily from the data) and was then deductive (based on key areas of interest covered by the interview questions).

The lead researcher (HQ) showed rigour and transparency (e.g., through reflective practice and being explicit about their position and with research decisions) and sought findings that would have practical implications. This was in addition to utilising a small team of independent researchers as ‘critical friends’ to offer alternative interpretations of the data and codes, credibility checks and support in the refinement of themes [22]. Reflexivity refers to the process of critically reflecting on the knowledge produced during the research process and the researcher’s role in producing that knowledge [21]. The lead researcher made notes of any preconceptions, impressions, ideas and early interpretations of the data throughout the research process and was conscious to make note of any personal biases. The research process contained unavoidable bias as the researchers on the evaluation team are *parkrun* participants. Reflective practice enabled the lead researcher to address these biases and consider preconceptions and expectations prior to conducting interviews (e.g., *what are your thoughts about parkrun?*); and to acknowledge biases and feed reflexive insight into the conduct of interviews (e.g., *what influence might my own assumptions have on the interview and findings generated?*). A similar approach to rigour and reflexivity has been used in previous *parkrun* research [15].

## Results

Fifteen *parkrun* Outreach Ambassadors representing 13 different LTC groups were interviewed. Participants varied across three categories; living with the LTC (*n* = 8), carer of someone with the LTC (carer; *n* = 3), specialist working in the field of the LTC (specialist; *n* = 4). Precise data about the length of time as Outreach Ambassador was not available, but it was intended that all interviewees were within 2 months of being appointed to their role. Demographic details of the sample (sex, age, occupation etc.) were not collected.

The main themes and sub-themes are described, with direct quotes taken from participants to help demonstrate the findings. Quotes are labelled with characteristics of the participant, but to protect confidentiality we have not provided details of the LTC they represent.

The analysis of the interviews resulted in four overarching themes and 13 subthemes that capture the perceptions of *parkrun* Outreach Ambassadors (see Table 1).

**Table 1:** Overview of themes and subthemes derived from interviews from *parkrun* Outreach Ambassadors

Theme	Theme label	Sample quotes
*Theme 1*	*Support for parkrunners with LTCs*	
Sub-theme 1a	*parkrun* has always been supportive of people with LTCs	*“there’s nothing formal, I would say but everybody was welcomed in one way or another” (P02, carer)*
Sub-theme 1b	Support from *parkrun* to engage those with LTCs could be more structured	*“There are instances where people who, whilst there’s no formal structure, have a huge amount of support. At the same time that doesn’t always happen, and there are parkruns that, through omission rather than by aim, don’t make it particularly easy for those with long-term conditions.” (P08, living with the LTC)*
Sub-theme 1c	The PROVE project is a strategic approach to inclusivity and publicity	*“It’s a great project in terms of what it’s trying to do. It hits on many different agendas and government initiatives trying to get the inactive active, which is great” (P15, specialist)*
*Theme 2*	*Perceptions of what success would look like for the PROVE project*	
Sub-theme 2a	Encouraging more *parkrun* participants who have LTCs	*“The ultimate success would be increased numbers of people with [health condition] taking part, that’s the overriding objective” (P09, carer)*
Sub-theme 2b	Creating a safe space for people with LTCs	*“A longer term aim is to influence healthcare professionals, politicians and third sector agencies like charities to promote … that parkrun is a safe place, to give doctors confidence to tell people go along.” (P11, living with the LTC)*
Sub-theme 2c	Being able to demonstrate sustainability of the PROVE project	*“in terms of a sustainable model, volunteers is a great way, because it’s cost-effective” (P15, specialist)*
*Theme 3*	*Contributors to PROVE project success*	
Sub-theme 3a	Being realistic about the potential for change	*“readjust and focus more on certain conditions where there’s a lower level of participation” (P15, specialist)*
Sub-theme 3b	Challenging perceptions of what *parkrun* is and who it is for	*“what we really need to be trying to get out there is, it is acceptable to come along to parkrun and walk” (P05, living with the LTC)*
Sub-theme 3c	Engaging with key stakeholders and advocacy groups	*“Being recognised by the health service and by the advocacy groups for the conditions is key” (P05, living with the LTC)*
Sub-theme 3d	Selecting Outreach Ambassadors with appropriate qualities	*“Passion. Persistence. Resilience. Stamina. Optimism” (P03, carer)*
*Theme 4*	*Anticipated challenges for the PROVE project*	
Sub-theme 4a	*parkrun* communication channels have limited reach	*“The limitations will potentially come from lines of communication not being robust enough” (P03, carer)*
Sub-theme 4b	Difficulty demonstrating impact	*“How do we know whether or not people have been encouraged to attend?” (P02, carer)*
Sub-theme 4c	Success is dependent on volunteers	*“It’s a massive piece of work, and to undertake it on top of your day job can be a bit difficult. So it’s about being realistic about what you can and can’t achieve in a short timescale” (P01, specialist)*

### Theme 1: existing support for parkrunners with LTCs

#### Sub-theme 1a: *parkrun* has always been supportive of people with LTCs

The Outreach Ambassadors were unanimous in their opinion that *parkrun* has always been supportive of people living with LTCs. This support was exemplified by the belief that *parkrun* has always been a welcoming and inclusive community for all. Comments from Outreach Ambassadors in reference to the supportive *parkrun* community included; *“parkrun has always been a very welcoming space”* (P11, living with the LTC); “*there’s nothing formal, I would say but everybody was welcomed in one way or another*” (P02, carer); “*actually being part of a community and just having that extra social aspect of it really made a difference*” (P13, living with the LTC); and *“there have been other people at parkrun who didn’t widely advertise the fact that they had conditions or disabilities, ranging from MS (Multiple Sclerosis) to Parkinson’s, but everybody has been very supportive of them”* (P05, living with the LTC). When asked how the support from *parkrun* differed to other sport or physical activity programmes, one Outreach Ambassador answered; “*I think it’s the removal of competitiveness: the idea that it’s not a race … at parkrun no-one really asks what your time is … so removing that pressure makes a huge difference, and that’s I think why it’s such an inclusive community*” (P13, living with the LTC).

#### Sub-theme 1b: support from *parkrun* to engage those with LTCs could be more structured

Whilst the *parkrun* organisation and *parkrun* community was deemed to be supportive of people with LTCs prior to the PROVE project, the Outreach Ambassadors had not witnessed any logistical support, formal policies or supportive resources offered by *parkrun* to people living with LTCs. One respondent suggested that prior to the PROVE project, there was; “*probably a lack of information about how we should support people, or how we should make it even more inclusive and encourage other people to come along as well*” (P02, carer). Whilst another Outreach Ambassador suggested that; “*there was some support, but only in ‘pockets’, it wasn’t done properly*” (P03, carer). It was generally believed that the level of support provided by *parkrun* for people with LTCs prior to the PROVE project lacked structure. For example:

*"There are instances where people who, whilst there’s no formal structure, have a huge amount of support. At the same time that doesn’t always happen, and there are parkruns that, through omission rather than by aim, don’t make it particularly easy for those with long-term conditions."* (P08, living with the LTC)

The consensus among those interviewed was that *parkrun*, whilst being inclusive already, had the potential to be more inclusive of those with LTCs; “*parkrun is inclusive but it still could be a lot more inclusive”* (P09, carer).

#### Sub-theme 1c: the PROVE project is a strategic approach to inclusivity and publicity

The PROVE project was valued as a targeted attempt at increasing physical activity opportunities for people with LTCs. One Outreach Ambassador said, “*It’s a great project in terms of what it’s trying to do. It hits on many different agendas and government initiatives trying to get the inactive active, which is great*” (P15, specialist). It was believed that the PROVE project provided a structured, strategic approach to inclusivity by enabling *parkrun* to raise awareness of LTCs and deliver support consistently across the community and across locations, with specific aims and objectives. The PROVE project was regarded as an opportunity to learn about the needs and desires of people living with LTCs so that *parkrun* can be aware and sensitive to these when providing physical activity opportunities that are appropriate for people from all backgrounds and abilities. For example, one Outreach Ambassador described how there are; “*certain things that we haven’t realised would stop people coming to parkrun. Like the crowd anxiety and some of the ways that you read out an event briefing*” (P13, living with the LTC).

In terms of the strategic priorities of PROVE, it was recognised that the PROVE project had two overarching aims; 1) to support existing *parkrun* participants living with LTCs by improving their experience and promoting continued participation, and 2) to encourage non-*parkrun* participants living with LTCs to take part in *parkrun*, as captured by the following:

*"Actually there are already lots of people with [health condition] who are doing parkrun. And we know that those aren’t our target audience because they’re already at parkrun and doing parkrun, although building a community for them has been part of our [PROVE] project. … So that’s great, but to be honest those aren’t our target audience, key audience. It is those people who aren’t parkrunning yet, to try and get them to come along"* (P07, living with the LTC)

Outreach Ambassadors also believed that the PROVE project gave *parkrun* the outlet for promotion and publicity to people with LTCs who may not be aware of *parkrun* or have not considered it an accessible activity. For example; “*the difference between now and prior to [the PROVE project] is there’s a real focus on encouraging people and making it easier for people to access parkrun, and then actively going out and publicising it to different communities*” (P02, carer). One Outreach Ambassador captured how the PROVE project differed from previous *parkrun* publicity techniques:

*"I would say that the PROVE project is completely counterintuitive to everything else that parkrun has done in the past. And what I mean by that is all parkruns appear to grow organically. They aren’t encouraged to advertise, they aren’t encouraged to go to clubs, they aren’t encouraged to go to the press, they aren’t encouraged to market; they grow organically through word of mouth. Whereas the PROVE project is actively making things easier for people with preconceived ideas or preconditions to say, look, why not come along and join. So it’s an interesting diversion and interesting change of strategy"* (P05, living with the LTC)

### Theme 2: perceptions of what success would look like for the PROVE project

Outreach Ambassadors were asked what they would deem successful outcomes for the PROVE project and this theme captures the most common responses.

#### Sub-theme 2a: encouraging more *parkrun* participants who have LTCs

Respondents believed that increased numbers of *parkrun* participants living with LTCs would be indicative of PROVE project success. One respondent commented; *“The ultimate success would be increased numbers of people with [health condition] taking part, that’s the overriding objective”* (P09, carer).

Whilst numerical evidence of increasing participation numbers was believed to be a desirable outcome, respondents acknowledged that being able to measure any increase in numbers of participants would be difficult, for example:

*"Ideally we would have something measurable that shows that we've supported people joining [parkrun] … but how we measure that is probably a challenge. It could be measured on how many [relationships with] organisations, charities are set up"* (P13, living with the LTC)

An alternative to having numerical evidence was to have qualitative or observational data in the form of stories, case studies and observations. For example, respondents believed a successful outcome for the PROVE project would be to witness more *parkrun* participants with LTCs at *parkrun* events and to be able to produce case studies to demonstrate participation, for example; “*more qualitative data, stories from people who said they didn’t do parkrun and it wasn’t for them and now they do it and think it’s for them”* (P11, living with the LTC) and; “*[success] is seeing more people with conditions at parkrun in different roles. Whether they’d be walking, running, in their wheelchairs, volunteering, or watching*” (P14, living with the LTC). An Outreach Ambassador had witnessed this type of success story on the Facebook group launched as part of the PROVE project to help *parkrun* participants with LTCs connect with others:

"*Someone had posted, 'I’ve been a member of the [Facebook] group for some weeks, my doctor told me I need to be more active, I’ve been really nervous about it, blah, blah, blah. But I went and did my first parkrun this weekend and it was brilliant. Thank you to the group for all the advice'. And she’d had lots of [encouragement], nothing very remarkable or practical, but really ‘oh come on, just do it, it’ll be fine, don’t worry about it’ sort of advice – and that was what she needed to get her off the sofa and out to parkrun, so fantastic. We’re building a community that will support that."* (P08, living with the LTC)

An Outreach Ambassador suggested that seeing increased numbers of *parkrun* participants who have LTCs would raise awareness among the rest of the *parkrun* community:

*"A by-product of all this [PROVE project] is just raising awareness in the community generally. So if you're somebody who's never met someone with [health condition] and suddenly you start seeing people with [health condition] taking part [in parkrun] it challenges stereotypes and prejudices and it will hopefully help people not make assumptions. But it's hard to measure that."* (P09, carer)

#### Sub-theme 2b: creating a safe space for people with LTCs to be active

Respondents believed that another indicator of PROVE project success would be to provide a supportive, safe space for people with LTCs; *“to provide a community and supportive space where people can get advice from like-minded people, support and encouragement”* (P11, living with the LTC). ‘Safe’ was defined in terms of *parkrun* events being welcoming and reassuring, as described:

*"The first stage of it is to make parkrun, the parkrun community … a safe space. And where people who have [health condition] or are at risk of [health condition] can turn up and feel comfortable. Where the people who are running the event or involved in the event or involved in the community feel comfortable with them being there, and basically lower those barriers to entry."* (P08, living with the LTC)

Respondents believed that a successful outcome would involve this ‘safe space’ becoming the norm at *parkrun* events and *parkrun* event teams having an awareness of LTCs, for example:

“*[Success would mean] that we eventually spread the word, and are so successful that everybody becomes so mindful and so aware of LTCs and the impact they can have, that it becomes almost normal and the setting up of new parkruns will take the needs of everybody into consideration*” (P01, specialist)

For one Outreach Ambassador, creating this ‘safe space’ involved; “*making sure that everything that we put out, or any campaign that we do is really well thought out, that the language is right and there’s nothing that’s going to trigger anything*” (P13, living with the LTC).

One way of promoting the safe space was thought to be through making contact with health professionals and establishing partnerships with advocacy groups and policy makers, who could endorse and promote *parkrun* to people living with LTCs:

*"A longer term aim is to influence healthcare professionals, politicians and third sector agencies like charities to promote … parkrun as a safe place, to give doctors confidence to tell people go along."* (P08, living with the LTC)

#### Sub-theme 2c: being able to demonstrate sustainability of the PROVE project

It was believed that success would be demonstrated if the impact of the PROVE project demonstrated longevity over time. An Outreach Ambassador believed that sustainability would involve the Outreach Ambassador role becoming redundant because; *“there won’t be a need for any Outreach Ambassadors as such, because everybody will think in the same way”* (P01, specialist). One Outreach Ambassador believed that being volunteer-driven facilitated sustainability, “*in terms of a sustainable model, volunteers is a great way, because it’s cost-effective*” (P15, specialist).

### Theme 3: contributors to PROVE project success

Respondents believed that the success of the PROVE project was dependent on the following factors; a) being realistic about the potential for change, b) challenging perceptions of what *parkrun* is and who it is for, c) engaging with key stakeholders and advocacy groups d) having Outreach Ambassadors with important qualities such as communication skills and experience of the LTC they represent.

#### Sub-theme 3a: being realistic about the potential for change

Success was believed to be dependent on having a realistic vision of the potential outcomes of the PROVE project and being realistic about the scale of the task given the limited resource available. Respondents acknowledged that it was important to be realistic about what the PROVE project could achieve, for example; *“what will give us the biggest impact from the least amount of intervention, small things that can have a big impact … not being overly ambitious and starting small”* (P11, living with the LTC). Likewise, an Outreach Ambassador talked about having realistic outcomes; “*parkrun is not a cure [for LTCs]* … *It doesn’t sort out these problems. But it does put people in an environment where they feel empowered and they feel able to exercise”* (P08, living with the LTC).

To overcome the large scale of the project, one respondent suggested that a realistic approach would be to prioritise certain LTC groups, “*readjust and focus more on certain conditions where there’s a lower level of participation*” (P15, specialist). Overall, respondents believed it was realistic to think about subtle changes to the *parkrun* organisation and environment, such as raised awareness among the *parkrun* community and *parkrun* event teams, for example:

*"I think we need to do some more stuff to raise awareness in the event teams and amongst the parkrun ambassadors about that. And I think that’s probably about as far as it goes. It would be lovely in an ideal world to have an amazing amount of resource of people who could parachute in to local events to support people with [health condition] if they wanted to run for the first time. But that doesn’t really fit the parkrun ethos, nor is it particularly practical."* (P08, living with the LTC)

Outreach Ambassadors believed that it was reasonable to use the PROVE project as an opportunity to learn, but that the impact would be subtle:

*"I see it [the PROVE project] as almost like a proof of concept pilot project, which is a vehicle for learning … analysing it, I'd like to see all that knowledge and data being used to learn … how we can learn to reach the difficult ones to get involved."* (P04, specialist)

*"I'd say the ethos of parkrun is that it doesn't change. It's so successful as a format and a concept I'm not sure that anybody wants it to change. I think it's really just about who gets involved, an awareness of the people who are involved … . the change is subtle."* (P04, specialist)

#### Sub-theme 3b: challenging perceptions of what *parkrun* is and who it is for

Respondents believed that success for the PROVE project was dependent on *parkrun* being accepted as an activity that is appropriate for everybody of all abilities, including people with LTCs, for example; “*we need to break down the stigma and preconceived ideas that people have got about what people living with LTCs can and can’t do”* (P01, specialist). It was believed that misconceptions about *parkrun* could be a deterrent to participation among some people with LTCs; *“people’s own perceptions put them off before they even arrive there, but you realise when you’re at parkrun it’s not too bad … I think fear is quite a lot of what people’s concerns are”* (P10, specialist). In relation to this, it was common for Outreach Ambassadors to refer to people’s “mind-sets” about physical activity for people with LTCs; *“Just trying to broaden people’s mind-sets. I think a lot of ladies with [health condition] have a fixed mind-set that exercise is not for them”* (P06, living with the LTC).

There was a belief that for the PROVE project to be successful, *parkrun* should be accepted as a walking event as well as a running event, for example; *“what we really need to be trying to get out there is, it is acceptable to come along to parkrun and walk”* (P05, living with the LTC). Some respondents felt the need to challenge the perception that *parkrun* was only for runners, for example; *“PROVE is about actively promoting that you can walk or jog it, despite the name ‘parkrun’“* (P11, living with the LTC). Another Outreach Ambassador reflected upon the preconceptions people can have about *parkrun*:

*"There’s this perception out there that people who run have got to be runners, they’ve got to be running about like Dave Bedford in singlets and be super fit. But I think that view has changed over the years with the jogging generation and the growth of things like the London Marathon, the Great North Run, the Race for Life. I think parkrun can continue that without having to go down the field of becoming 'parkwalk' for instance. I think if it became 'parkwalk' it would put the runners off, and actually it did start with the runners. The clue’s in the name: parkrun. But we do say welcome to all. So I would like to see that we still have people who are not afraid to come up and run, but people who aren’t afraid to come and walk too."* (P05, living with the LTC)

#### Sub-theme 3c: engaging with key stakeholders and advocacy groups

Outreach Ambassadors believed that the success of the PROVE project was dependent on engagement with key stakeholders and advocacy groups associated with different LTCs, believing that this would help reach a wide range of people in the general population; “*that’s how we can actually get that sort of message across*” (P02, carer). Another Outreach Ambassador agreed that engagement with advocacy groups such as national charities was the key to broadening the PROVE project’s reach; *“I think that’s the key to opening parkrun up to more people”* (P06, living with the LTC).

An Outreach Ambassador suggested that engagement with key stakeholders might depend on *parkrun* being recognised and valued as a health intervention, for example; *“being recognised by the health service and by the advocacy groups for the conditions is key”* (P05, living with the LTC). Another Outreach Ambassador suggested that working alongside healthcare professionals would be advantageous for ensuring the right messages are being communicated to people with LTCs; *“The last thing I want to do is turn up and talk to someone with a heart condition where running is the last thing that they should be doing … we need to work more hand in hand with the professionals”* (P05, living with the LTC).

#### Sub-theme 3d: selecting outreach ambassadors with appropriate qualities

Respondents were asked to describe the qualities needed to be successful Outreach Ambassadors. The majority described a passion for change and making a difference. Communication and experience of the condition were also believed to be important. The following descriptions were provided by the Outreach Ambassadors:

“*To be a good communicator, to be a good listener, to be aware of the developments that are happening around you, and to not be confined by your own thinking, be willing to listen to what other people have got to say*” (P01, specialist)

*“Passion. Persistence. Resilience. Stamina. Optimism”* (P03, carer)

*"Really good communication skills. Especially when you're putting things on a [Facebook] group, it needs to be worded carefully so its emotive but it doesn’t make people feel guilty … being organised, having good empathy, being a team player, having a good understanding of the aims of the project … action planning, reflective, being aspirational in terms of what you hope to achieve"* (P06, living with the LTC)

*"Empathy with the condition and an awareness of the limits of the role" … “non-prejudice”* (P10, specialist)

*"nothing about us without us … it's about people with the condition or disability saying this is my experience, this is what I need, I'm an expert by virtue of my experience and I think that's fundamentally important to the PROVE project … I don't think it would work without that"* (P11, living with the LTC)

It was believed that the autonomy given to Outreach Ambassadors was an important driver for PROVE project success. One respondent suggested, “*It’s not a top down approach, micro-managing the volunteers … it’s very much a free sort of thing, to come and do as and when you can on the project and do what you can*” (P15, specialist).

### Theme 4: anticipated challenges for the PROVE project

The Outreach Ambassadors recognised a number of challenges for the PROVE project. Whilst the PROVE project was considered to have realistic aims and objectives that align well with the overall *parkrun* strategy, it was considered a large, challenging task; “*it’s challenging and each [condition] group will have different challenges*” (P02, carer). One Outreach Ambassador described the size of the challenge as “phenomenal”:

*"I think it's just an absolutely phenomenal challenge. You know, the NHS and the government are trying to do this and have got millions of pounds and then it feels like there are us parkrun Outreach Ambassadors, … it does feel like a massive, massive task. But then parkrun is a massive thing."* (P07, living with the LTC)

The subthemes capture the main challenges perceived by respondents that relate to methods of communication, difficulty demonstrating impact and the dependency on volunteers.

#### Sub-theme 4a: *parkrun* communication channels have limited reach

The Outreach Ambassadors believed that the word-of-mouth publicity of *parkrun* has resulted in internal communications among like-minded people, for example:

*"It’s easy to get like-minded people involved. It's getting people, the people who aren't of the same kind of mind-set involved who could benefit, so maybe there's a challenge there … I'm not sure that the objectives of the PROVE project are going to touch the people that we as a society need to reach"* (P04, specialist)

The main belief among the Outreach Ambassadors was that for the PROVE project to be successful, its communication, reach and engagement “*needs to go outside of parkrun”* (P10, specialist) and reach the non-*parkrun* community.

There was also reference to the communication of PROVE project activity within *parkrun*, with some Outreach Ambassadors suggesting that the project’s aims and reach are not well understood among existing *parkrun* participants, for example; “*if you’re a normal parkrunner you might only get snippets of the different conditions and not realise that we’ve got this entire [PROVE] programme that supports lots of different ones*” (P13, living with the LTC). Communication was particularly important for the Outreach Ambassador for deaf and hard of hearing, who believed that communication barriers could be a problem; “*The limitations will potentially come from lines of communication not being robust enough*” (P03, carer).

#### Sub-theme 4b: difficulty demonstrating impact

Another challenge perceived by the Outreach Ambassadors was in demonstrating the impact of the PROVE project. As demonstrated in Theme 3, respondents believed that to be successful, the PROVE project would need to demonstrate an increase in the numbers of *parkrun* participants with LTCs participating. Yet respondents were unsure whether this is possible; “*how do we know whether or not people have been encouraged to attend?”* (P02, carer).

Outreach Ambassadors also perceived the challenge of knowing how much of an increase in participation would be regarded a success; “*One of the challenges is - we’ve got some baseline figures, but it’s going to be hard to actually quantify it”* (P09, carer). Another Outreach Ambassador described their concern in quantifying impact:

*"I don’t know what an acceptable number of new parkrunners would be, if we get 10 new people, would that be an achievement or 100 or … ? … If at the end of it we go, well, actually, do you know what, we know that we got 10 people with [health condition] to be more active, and we give ourselves a big pat on the back, but what I’m not sure on is what is considered a success?"* (P07, living with the LTC)

Given the challenges of demonstrating impact numerically, the respondents believed that impact would need to be demonstrated in different ways, particularly, “*what the participants feel, their life changes, actually how it’s impacted and changed their lives completely … that side of stuff is very important to measure … the life outcomes, life skills*” (P15, specialist).

#### Sub-theme 4c: success is dependent on volunteers

Another challenge cited by the Outreach Ambassadors was the limitations associated with the PROVE project being dependent on the work of volunteers for its delivery and the realisation that; “*there’s only so far you can take the voluntary sector*” (P05, living with the LTC). Another respondent explained; “*It’s a massive piece of work, and to undertake it on top of your day job can be a bit difficult. So it’s about being realistic about what you can and can’t achieve in a short timescale”* (P01, specialist). The same Outreach Ambassador who believed that the volunteer-driven approach was the most sustainable model also believed that this introduced, “*limitations of what it might achieve*” (P15, specialist) because of other responsibilities and time constraints.

Some respondents were concerned about the extra burden that might be placed on volunteering *parkrun* event teams if the number of *parkrun* participants living with LTCs increased significantly. One respondent suggested that success of the PROVE project might depend on upskilling volunteer event teams (the *parkrun* volunteers delivering the events in local communities), “*so you could give them some skills to then help and support engage people with the health condition or disability in parkruns. So they are upskilled enough to then support people*” (P15, specialist). However, there was evidence of concerns about placing too much burden on volunteers, for example; *“everything we do is with the awareness that event teams are volunteers and we can’t place too much burden on them”* (P11, living with the LTC) and also; *“it’s all been done on a low level volunteer basis … we’re putting a big onus on the volunteers and a big weight on their shoulders … being asked to be welcoming to all and they may not be able to handle it”* (P05, living with the LTC).

## Discussion

Physical activities with a focus on community and shared interest have been recommended [3], but practical examples of approaches to promote physical activity among people living with LTCs are needed [23]. *parkrun* launched the PROVE project to promote participation and support the experience of *parkrun* among people living with LTCs. This research interviewed *parkrun* volunteer Outreach Ambassadors to explore the perceptions of the PROVE project for people living with LTCs. The findings support previous research that has shown how *parkrun* is regarded as an inclusive community physical activity opportunity [13–15]. It also demonstrates the perceived benefits that *parkrun’s* PROVE project has had for people living with LTCs. According to the perspective of the Outreach Ambassadors interviewed, the PROVE project was welcomed by *parkrun* participants who have LTCs and praised for enabling a more structured and consistent approach to welcoming people to *parkrun* and supporting their positive experience.

According to the Outreach Ambassadors, the PROVE project had the potential to enable *parkrun* to be a safe and welcoming space for people with LTCs to engage in physical activity and volunteering. *parkrun*’s ability to create social support networks in communities supports Public Health England’s [3] request for social network approaches that focus on strengthening community and social support between people, via collective or community activities. Rimmer and Marques [9] outlined the urgent need for approaches that integrate people with LTCs into existing community-based physical activity services. Guidelines for the implementation of community-based health promotion programmes for people with disabilities state that communities should provide socially engaging physical activity environments that will allow people with LTCs to engage in physical activity with other community members [24]. The guidelines recommend that disability and non-disability service providers must work together to form inclusive health coalitions that represent the physical activity needs of community members with LTCs. The communities created by *parkrun* and the PROVE project, either in real life or online (i.e., via Facebook support groups) were regarded as important for creating social networks and breaking down barriers to physical activity and/or volunteering for people living with LTCs. This supports previous research demonstrating the mental health benefits of being identified as part of the *parkrun* community [15]. The current findings demonstrate the importance of people living with LTCs feeling part of a social community and the potential role that *parkrun* could have in offering inclusive physical activity and volunteering opportunities (i.e., *parkrun’s* ‘social capital’). The role of ‘social capital’ in shaping participation in *parkrun* has been explored previously [25] and further investigation is needed into how social relationships as sources of support may promote participation among people living with LTCs. The PROVE project provided *parkrun* with the opportunity to engage with people living with LTCs to better understand their needs and desires and take these into consideration when designing and delivering targeted interventions to promote *parkrun* to wider audiences.

According to the Outreach Ambassadors interviewed, success of the PROVE project was believed to be dependent on being realistic about the potential for the project to bring about measurable change given the financial, time and resource limitations of the volunteer sector. Success was also deemed to be dependent on *parkrun* successfully challenging misconceptions that *parkrun* is for runners only. Previous *parkrun* research presents examples of how acceptance and provision for individuals with visual impairments and welcoming groups of Nordic walkers demonstrates the potential for parkrun to attract ‘non-traditional’ populations [13, 26], but the current findings suggest that more can be done to communicate that *parkrun* is welcoming and inclusive to all. Guidelines for health promotion programmes for people with disabilities suggest that opportunities should be socially, behaviourally, and environmentally accessible [24]. Whilst the current study suggested that *parkrun* is deemed a ‘safe space’ and physically accessible for some people with LTCs, which supports previous findings [15], lack of accessibility might be perceived by others. Attempts should be made to ensure that events like *parkrun* and their communication channels are accessible for all, but that they are perceived as welcoming and appropriate for all.

The Outreach Ambassadors interviewed identified a number of challenges for *parkrun* in delivering the PROVE project. Outreach Ambassadors had concerns about communication barriers, which could be pertinent to some LTCs more than others. For example, individuals who are deaf or hard of hearing will not necessarily learn about *parkrun* from channels such as social media and newsletters, thus communications involving talking newspapers or British Sign Language are important considerations [24]. Similarly, people with learning disabilities may have communication barriers requiring accessible documents and easy read information. Thus, to optimise the success of the PROVE project and similar initiatives it is important for *parkrun* to establish communication methods that reach widely both within and outside the *parkrun* community. To facilitate this, forming partnerships with key advocacy groups and charities at regional and national level would enable *parkrun* to reach the non-*parkrun* community with messages and promotion. Prescribing *parkrun* has been formally recognised in the UK by the Royal College of General Practitioners (RCGP). This new partnership with RCGP involves *parkrun* events linking with their local GP practice who become certified as ‘*parkrun practices*’ with clinical champions referring *parkrun* to patients and their cares [27, 28]. It would be beneficial for events like *parkrun* to work with healthcare professionals specialising in LTCs to ensure that messages are appropriate for different health conditions and to encourage the ‘social prescription’ of physical activity to people living with LTCs.

There are wider implications of the findings for policy makers and physical activity providers for the design and implementation of inclusive community-based activity opportunities for people with LTCs. The results provide an opportunity for shared learning and for *parkrun* to demonstrate how approaches to promoting physical activity among people with LTCs work on a wide scale. There is a need to; i) be clear about how to measure the impact of such interventions, ii) be realistic about the potential to make significant change to health and behaviour, especially if implementation is dependent on the voluntary sector, iii) meet the needs of the communities being targeted, iv) ensure good communication channels that reach the target audience and, v) recruit Outreach Ambassadors, champions or community role models that have qualities such as passion and experience of the LTC. *parkrun*’s PROVE project could be an exemplar of how community-based social support networks can be used to support people with LTCs to self-manage their condition and overcome barriers to physical activity. The research team plan to disseminate the findings from the PROVE project evaluation to enable important learnings to be shared.

### Evaluation

The results of this research should be considered in light of the following methodological issues. The findings reflect the views of self-selecting *parkrun* Outreach Ambassadors only and therefore might not be representative of the views of *parkrun* participants who have LTCs or the wider population of people living with LTCs. The experience of *parkrun* participants who may have had a negative experience of *parkrun* has not been captured in this study. A similar critique has been highlighted in previous *parkrun* research [13]. A limitation of the methodology is that the same researcher, who is a registered *parkrun* participant, conducted the interviews and analysed the data, so the findings should be interpreted with potential for bias in mind. However, the researcher’s familiarity with *parkrun* was believed to facilitate the conduct of the interviews (i.e., in establishing rapport) and the researcher engaged in reflective practice to bring awareness to any preconceptions, beliefs and opinions about *parkrun* and the advantages and disadvantages that brought to the analysis of data. Furthermore, the analysis of data utilised a group of independent ‘critical friends’ as a research tool to help refine themes. The decision to include participants with different types of experience (i.e., people living with, caring for someone or working within the LTC) was a pragmatic decision given that all Outreach Ambassadors appointed by *parkrun* were invited to interview. However, the current research does not explicitly explore differences in perceptions by respondent category. Although this introduces methodological inconsistency in participant type, the researcher was careful to check for contrasting opinions in the analysis of data.

## Conclusions

The need for inclusive community physical activity opportunities that are appropriate for people with LTCs, delivered in a structured manner and communicated appropriately are a priority. *Parkrun*’s PROVE project has the potential to ensure that *parkrun* remains an inclusive and welcoming environment for people with LTCs to engage in physical activity and/or volunteering. The findings from this study have important implications for policy makers and physical activity providers wishing to design, deliver and evaluate community-based physical activity opportunities for people with LTCs.

## Data Availability

The anonymised datasets used and/or analysed during the current study are available from the corresponding author on reasonable request.

## Abbreviations

LTC(s): Long term condition(s)
PROVE: *parkrun* running or volunteering for everyone
RCGP: Royal College of General Practitioners
